# Quality Evaluation and Heat and Mass Transfer Mechanism of Microwave Vacuum Drying of *Astragalus* Roots

**DOI:** 10.3390/foods13193075

**Published:** 2024-09-26

**Authors:** Yuanman Yue, Qian Zhang, Guojun Ma, Fangxin Wan, Zepeng Zang, Yanrui Xu, Futai Kang, Xiaopeng Huang

**Affiliations:** 1 College of Mechanical and Electrical Engineering, Gansu Agricultural University, Lanzhou 730070, China; 2 College of Mechanical and Electronic Engineering, Northwest A and F University, Yangling 712100, China; 3 Transportation Department of Education, Aksu Prefecture Kuqa Secondary Vocational and Technical School, Kuqa 841000, China

**Keywords:** *Astragalus*, microwave vacuum drying, drying kinetics, heat and mass transfer mechanism, quality analysis

## Abstract

In this research, the objective was to optimize the drying process of *Astragalus* by investigating the effects of microwave vacuum drying parameters, including temperature (30, 35, 40, 45, and 50 °C) and slice thickness (2, 3, 4, 5, and 6 mm). In addition, utilizing COMSOL 6.0 finite element analysis software, we delved into the distribution of heat and moisture during the drying process. The results revealed that drying temperature played a significantly greater role than slice thickness in determining the drying dynamics. The thermal and mass transfer mechanism indicated that the whole drying process conforms to the microwave radiation mechanism and the basic principle of electromagnetic heating. In the case of low temperatures and thinner slice sizes, the more polysaccharide content was retained; The total phenol content peaked when the slice thickness was 5 mm; The increase of slice thickness was not conducive to the retention of total flavonoids content. The potent antioxidant capacity was detected at a temperature of 40 °C, with slice thickness having a negligible effect on this capacity; Low temperatures were beneficial for the preservation of active ingredients. Compared with the scanning electron microscope, the structure appeared more uniform at a temperature of 50 °C. Based on the analysis of the kinetic characteristics of microwave vacuum drying of *Astragalus* and the quality achieved under various drying conditions, the results of the study can provide valuable guidance for controlling the quality of microwave vacuum drying of *Astragalus* under different drying requirements.

## 1. Introduction

*Astragalus* is a legume *Astragalus membranaceus* (Fisch.) Bge. var. *mongholicus* (Bge.) Hsiao or *Astragalus membranaceus* (Fisch.) Bge. [1]. *Astragalus*, a revered medicinal herb, has been a cornerstone in therapeutic practices across China and various Asian nations for millennia. Its dried roots, celebrated for their healthful and strengthening properties, have been meticulously documented in numerous scholarly works [2,3,4,5]. *Astragalus* is a favored by consumers of medicinal materials, as it contains a large number of bioactive compounds as well as a large number of trace elements required by the human body, such as selenium and zinc, among 14 others. These elements bestow upon *Astragalus* its notable medicinal and economic significance [6,7]. Fresh raw matter has high water content and is abundant in carbohydrate compounds. If the drying process cannot be completed quickly after harvest, the quality of the medicinal materials will be greatly reduced. Therefore, there is an urgent need to propose feasible processing and preservation technology to extend its shelf life, reduce economic losses and environmental losses, stabilize the price of traditional products and increase their commercial value.

In the realm of *Astragalus* processing, the prevalent methods for drying the herb’s slices encompass vacuum drying, hot air drying, and vacuum freeze drying. However, significant challenges remain in achieving uniform drying and improving process efficiency. Improving these areas is crucial to ensure that the dried product retains its optimal medicinal qualities while making the drying process as effective and economical as possible [8,9,10]. Microwave vacuum drying utilizes electromagnetic waves to enhance friction between water molecules, leading to volumetric heating. Compared to other methods, it boasts faster heating and lower energy consumption. The vacuum environment accelerates water evaporation, enhances heat transfer to the material surface, and reduces thermal stress at sub-atmospheric pressures, thereby preventing the harmful effects of heat and oxidation on sensitive nutrients. Consequently, this technique, widely used in food processing, significantly minimizes the loss of heat-sensitive components [11,12,13]. Purple tuber sweet potatoes dried using microwave vacuum and hot air, and it was discovered that microwave vacuum drying resulted in much shorter drying times and considerable benefits in terms of color preservation, rehydration, antioxidant content, and total phenolic content [14]. The shelf life of the material was found to be increased by microwave vacuum drying in the evaluation of the physicochemical parameters of sweet potato chips [15]. In order to achieve the best process parameters for uniform drying, preserving the best color, and preventing burning, one researcher chose potato as the raw material and optimized the microwave vacuum drying method for potato [16]. The use of vacuum had a greater impact on heating and drying efficiency as the moisture content dropped, according to research on microwave vacuum drying of banana slices [17]. Hamza compared the effects of different drying techniques on the drying kinetics and quality of orange slices, and found that microwave vacuum drying technology significantly improved the drying rate and effective moisture diffusivity of orange slices [18]. In recent years, scholars have also explored the microwave vacuum drying characteristics of Dendrobium ferruginum [19], ginseng slices [20], Angelica [21] and other herbs. Despite these, challenges arise due to varying material shape and uneven moisture distributions on surfaces, often resulting in uneven heat and moisture loss, and potential over-heating. To overcome these, our research institute has developed a custom-designed, spiral-type microwave vacuum dryer. The device intertwines microwave energy with the rotating drum, which, when it rotates vertically along a horizontal axis, systematically raises trays from beneath the microwave source. This intermittent drying method enhances uniformity, harnessing the benefits of microwave vacuum drying. It ensures that intricate crop materials, with their heightened sensitivity to heat, receive a more controlled environment for drying. The drying equipment used in this experiment has passed the microwave leakage test and is harmless to the human body. In addition, the equipment does not produce toxic gases during operation, has no pollution to the environment, and has good environmental protection performance.

The focus of this study is to explore the behavior pattern of microwave vacuum drying of *Astragalus* slices under multiple drying conditions, including different temperatures and thicknesses. At the same time, we are also committed to in-depth evaluation of its quality after drying. Through precise numerical simulation, we try to reveal the specific effects of microwave vacuum drying process on temperature and moisture content. The goal is to provide scientific basis and advanced technology support for microwave vacuum drying process.

## 2. Materials and Methods

### 2.1. Test Material

Fresh samples used in this experiment were procured from Min County Guiqi Ginseng Growers Cooperative (Dingxi, China). Following acquisition, they were placed under refrigeration at 4 °C. The samples were taken out 1 h before the experiment. After the temperature reached room temperature (22 ± 1) °C, the samples without mechanical damage and obvious branches were selected for the experimentas. In accordance with the AOAC standard procedure, we used fresh *Astragalus* as a sample and conducted 24-h insulation treatment at 105 °C. The average water content was accurately measured to be 55.5% [22].

### 2.2. Test Equipment

**Figure 1:**
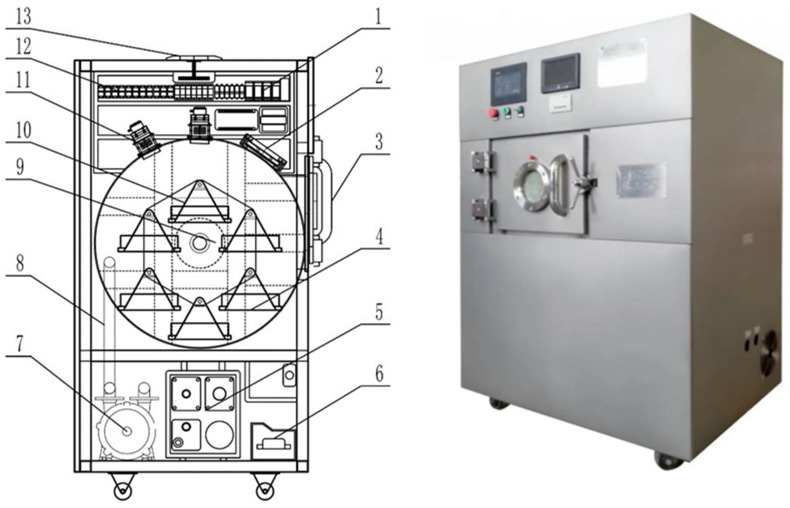
Structure diagram of microwave vacuum dryer. 1. PLC control box; 2. ventilation port; 3. Furnace door; 4. Dynamic material tray; 5. Vacuum pump auxiliary part; 6. Circuit protection part; 7. Vacuum pump; 8. Vacuum tube; 9. Motor; 10. Pallet receiving frame; 11. Microwave generator; 12. Circuit controllers; 13. Cooling holes.

The microwave vacuum dryer used in the test was jointly developed by the School of Mechanical and Electrical Engineering of Gansu Agricultural University (Lan Zhou, China) and Tianshui Shenghua Microwave Technology Co., Ltd. (Tian Shui, China). The structure is shown in Figure 1. The equipment mainly includes main box, microwave radiation system, vacuum system and circuit control system. The microwave radiation system consists of two microwave generators. The vacuum auxiliary system consists of vacuum pump auxiliary part, vacuum pump and vacuum tube. The circuit control system consists of a PLC control system and a circuit protection system. Its microwave power is 3 kW, rotational speed range: 0~50 Hz, temperature range: 30~150 °C, adjustable vacuum range: −0.080~−0.060 MPa. There are six material trays in the drying chamber. When the equipment is working, the rotation of the material tray driven by the motor is close to the microwave generator in turn, which can improve the uniformity of the drying process. The auxiliary part of the vacuum pump controls the operation of the vacuum pump to make the internal environment of the box reach a vacuum state, reduce the boiling point of water, and achieve the effect of low temperature drying.

### 2.3. Experimental Methods

**Figure 2:**
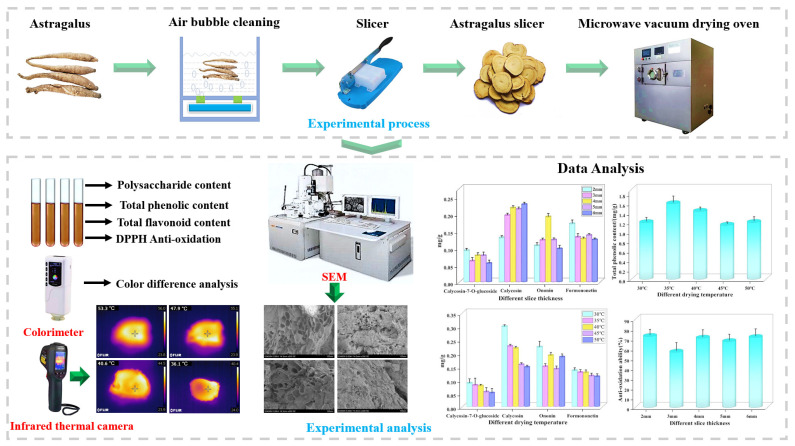
Microwave vacuum drying and quality detection of *Astragalus* schematic diagram.

The variables in our microwave vacuum drying assessment of *Astragalus* were sliced thickness and temperature. Combined with the preliminary experiment and equipment parameters, tests were carried out at five drying temperature levels (30, 35, 40, 45 and 50 °C) and five slice thickness (2, 3, 4, 5 and 6 mm). Before the experiment, the *Astragalus* with the same thickness and higher moisture content was selected and cleaned. The homemade slicing machine was used to cut the material to the required thickness under different conditions, and the slices were weighed to 120 g ± 2 g. After adjusting the drying oven to the preset temperature, the materials were placed into the chamber of the drying equipment for drying test. The weight and moisture content of the slices were recorded by every 8 min. When the moisture content drops below the safe moisture content (10%), the trial was halted. The comprehensive experimental sequence, depicted in Figure 2, encapsulates all steps involved in this process.

### 2.4. Test Index Determination

#### 2.4.1. Determination of Moisture Content

(1) $$X=\frac{M_{t}-M_{d}}{M_{d}}$$

where: X denotes dry basis moisture content, %; *M_t_* denotes the weight of dried product at moment *t*, g; *M_d_* denotes the dry weight of dried product, g.

#### 2.4.2. Determination of Drying Rate

(2) $$DR=\frac{M_{t_{2}}-M_{t_{1}}}{t_{2}-t_{1}}$$

where: DR denotes the drying rate, g/s; *M_t_*_2_, *M_t_*_1_ denotes the weight mass of dried product slices at moments *t*_2_ and *t*_1_, g; *t*_2_ − *t*_1_ denotes the time interval between the two weighings, min.

#### 2.4.3. Determination of Moisture Ratio

(3) $$MR=\frac{M_{t}-M_{e}}{M_{c}-M_{e}}$$

where: *M_t_* denotes the dry basis moisture content of dried product at moment *t*, %; *M*_c_ denotes the initial moisture content of dried product, %; *M_e_* denotes the moisture content of dried product dried to equilibrium, %.

Since the equilibrium water content of *Astragalus* is small, the simplified equation chosen here is:

(4) $$MR=\frac{M_{t}}{M_{c}}$$

### 2.5. Numerical Simulation and Verification

#### 2.5.1. Microwave Drying Characteristics

Microwave is a kind of electromagnetic wave with very short wavelength. Its wavelength is between 1 mm–1 m and its frequency is between 300 GHz–300 MHz. Their utility in heating is hinged on two key conditions when employed: equal dissemination of microwave energy within the heating chamber to avoid uneven heating, and a well-matched impedance between the waveguide and the device cavity to prevent device damage. In material microwave drying, variations in properties, such as microwave transmittance, reflection, and absorption rate due to their unique characteristics, significantly influence drying behaviors. A thorough investigation of these materials’ drying characteristics under microwave exposure allows for enhancing the drying speed and uniformity effectively.

#### 2.5.2. The Characteristic of the Electromagnetic Wave Propagates

The principle of microwave drying and radio frequency drying are conceptually parallel. When microwave drying is performed on different substances, the amount of microwave absorbed will reflect the drying efficiency of the substance. The device contains a waveguide, responsible for emitting microwave waves into the dried material, thereby heating it. The heating process follows Maxwell’s formula.

(5) $${\nabla_{\mu_{r}}}^{-1}\left(\nabla E\right)-\left(\frac{2\pi f}{c}\right)\left(\varepsilon^{\cdot }-j\epsilon \right)E=0$$

where: $\mu_{r}$ denotes the relative permeability; *E* denotes the electric field strength, V/m; *c* denotes the speed of light, 3.0 × 10^8^ m/s; $\varepsilon^{\cdot }$, *ε* denotes the dielectric constant and dielectric loss factor; *f* denotes the electromagnetic field frequency, Hz.

#### 2.5.3. Heat and Mass Transfer Control Equation

The essence of microwave heating is electromagnetic induction heating, which is used to produce Joule heating. It can be expressed by the following formula:

(6) $$\alpha \frac{\tau E}{\tau F}+\nabla \times \left(\mu_{0}^{-1}\mu_{r}^{-1}F\right)=J$$

where: *F* denotes the magnetic flux density, *E* denotes the vector magnetic potential, and *J* denotes the bulk current density.

The microwave drying process of *Astragalus membranaceus* includes two aspects: electromagnetic and heat conduction. There is a two-way coupling between the two. Therefore, the heat transfer equation in the microwave drying process of *Astragalus membranaceus* can be expressed as:

(7) $$\rho C_{p}\frac{\partial T}{\partial t}+\nabla \cdot \left(-k\nabla T\right)=Q$$

where: *ρ* denotes the vacuum density, kg/m^−3^; *q* denotes the microwave energy absorbed by the material of *Astragalus*, W·m^−3^; *k* denotes the thermal conductivity, W·m^−1^·K^−1^; $C_{p}$ denotes the specific heat capacity, J·kg^−1^·K^−1^.

In the process of microwave drying astragalus, not only involves the transformation of heat, but also accompanied by the diffusion of water. Then, the mass transfer equation of microwave drying *Astragalus* can be expressed as:

(8) $$\frac{\partial c}{\partial t}+\nabla \cdot \left({-D}_{eff}\nabla c\right)=0$$

where: $D_{eff}$ denotes the effective diffusion coefficient of water, m^2^·s^−1^; *c* denotes the instantaneous water concentration mol·m^−3^.

#### 2.5.4. Dielectric Properties of Samples

The dielectric property of the material refers to the absorption capacity of the material to microwave radiation when the material is heated by microwave. The dielectric constant of the material reflects the material’s ability to constrain the charge, which is directly reflected in the material’s ability to absorb light and heat, and the dielectric properties of the material vary with the type of material. At the same time, the dielectric constant of the material will change dynamically with the change of the internal temperature and moisture of the material, and the dielectric properties of the material are the most direct characterization of the microwave absorption, conversion efficiency and thermal behavior of the material. The complex dielectric constant refers to the comprehensive effect of dielectric coefficient and loss coefficient on the microwave absorption performance of the material. The complex dielectric constant is used to characterize the microwave absorption performance of the material.

(9) $$P=2\pi fE^{2}\varepsilon$$

where: *P* denotes the microwave absorption power, W/m^3^; *f* denotes the microwave frequency, Hz; *E* denotes the electric field strength, V/m; *ε* denotes the dielectric constant and dielectric loss factor.

#### 2.5.5. Construction of Numerical Model

Based on the above microwave drying principle, the microwave drying model of *Astragalus* was constructed by COMSOL 6.0 finite element analysis software. In order to reduce the time of subsequent simulation, the microwave equipment model was simplified, and the sample model of *Astragalus* with a diameter of 8 mm and a thickness of 4 mm was established. In order to improve the accuracy of the simulation results, the following assumptions are made to the model:

(1) The initial temperature and medium of *Astragalus* are uniform and isotropic in its interior;
(2) Electromagnetic wave is transverse wave or flat wave without omission;
(3) The gas phase in the microwave drying equipment is in an ideal state;
(4) It is assumed that *Astragalus* did not deform during microwave drying;
(5) *Astragalus* conforms to the linear constitutive relationship.

**Figure 3:**
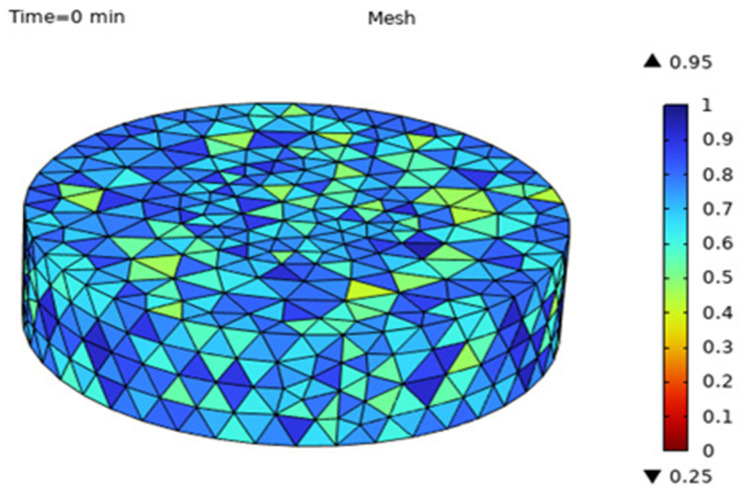
Grid division of Astragalus samples.

After setting the simulation parameters, the grid division is carried out. The results of the grid division of the Astragalus sample include 49,184 domain units, 4848 boundary units and 392 boundary units. The mesh quality is shown in Figure 3.

**Figure 4:**
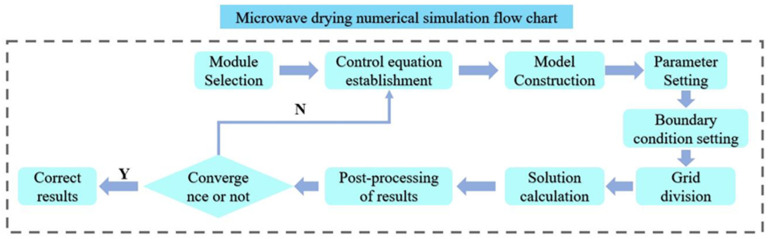
Numerical simulation flow chart.

After meshing, the simulation is solved. The numerical simulation process is shown in Figure 4.

### 2.6. Quality Index Measurement

#### 2.6.1. Preparation of Extracts

A slight change was made in the research methods of Yue et al. [23]. Weigh 0.5 g of dried sample powder and add it to 25 mL of anhydrous ethanol at a ratio of 1:5 (m/V) in a 50 mL centrifuge tube. Shake for 48 h on a constant temperature shaker (120 r/min) in darkness. Then centrifuge for 10 min (set parameters 4 °C, 5000 r/min) and take the supernatant. The filtrate was stored at 4 °C. Polysaccharides, total phenols, total flavonoids and antioxidant capacity were measured by using the extract.

#### 2.6.2. Determination of Polysaccharide Content

The polysaccharide content was determined by phenol sulfuric acid procedure [24]. The standard curve was obtained by using glucose standard solution as the control standard (With the concentration of sugar solution as the independent variable x and the absorbance as the dependent variable y): y = 0.00835x − 0.163 (r^2^ = 0.99355). The polysaccharide content was calculated according to Equation (10):

(10) $$\mathrm{Polysaccharide}\;\mathrm{content}=\frac{V_{Sample}C_{Glucose}}{V_{1}M}$$

where: *C_glucose_* denotes the content of glucose in the sample determination tube, μg; *V*_1_ denotes the volume of sample extraction solution for aspiration determination, mL; *V_sample_* denotes the total volume of sample extraction solution, mL; *M* denotes the mass of sample in the extraction solution, g.

#### 2.6.3. Determination of Total Phenolic Content

The total phenolic content was determined according to the Folin-Ciocalteu reagent method [25]. The standard curve was developed using gallic acid as the control standard (Gallic acid concentration was used as the independent variable x and absorbance as the dependent variable y): y = 0.0498x + 0.5051 (r^2^ = 0.9905). The total phenolic content was calculated according to Equation (11):

(11) $$\mathrm{Total}\;\mathrm{phenolic}\;\mathrm{content}=\frac{V_{Sample}C_{Gallic\;acid}}{V_{2}M}$$

where: *C_gallic acid_* indicates the content of gallic acid in the sample determination tube, μg; *V*_2_ indicates the volume of sample extraction solution for aspiration determination, mL; *V_sample_* indicates the total volume of sample extraction solution, mL; *M* indicates the mass of sample in the extraction solution, g.

#### 2.6.4. Determination of Total Flavonoid Content

The NaNO_2_-Al (NO_2_)_3_-NaOH method was used to determine the total flavonoid content [26]. The total flavonoid content was standardized by using catechin as the control standard to obtain the standard curve (With catechin concentration as the independent variable x and absorbance as the dependent variable y): y = 0.0053x + 0.037 (r^2^ = 0.9984). The total flavonoid content was calculated according to Equation (12):

(12) $$\mathrm{Total}\;\mathrm{flavonoid}\;\mathrm{content}=\frac{V_{Sample}C_{Catechin}}{V_{3}M}$$

where: *C_catechin_* indicates the amount of catechin contained in the sample determination tube, μg; *V*_3_ indicates the volume of sample extraction solution for aspiration determination, mL; *V_sample_* indicates the total volume of sample extraction solution, mL; *M* indicates the mass of sample in the extraction solution, g.

#### 2.6.5. Determination of Antioxidant Properties

Total antioxidant capacity was determined using the DPPH method [27]. The antioxidant capacity was calculated according to Equation (13):

(13) $$\mathrm{Total}\;\mathrm{antioxidant}\;\mathrm{capacity}=\frac{A_{Without\;sample}-A_{Sample}}{A_{Without\;sample}}$$

where: *A_sample_* is the absorbance value of the sample solution; *A_without sample_* is the absorbance value without the sample solution.

### 2.7. Determination of Active Ingredients

#### 2.7.1. Chromatographic Conditions

Chromatographic columns: Agilent SB-C18 (Merck, Darmstadt, Germany) (250 mm × 4.6 mm, 5 μm); Mobile phase: Acetonitrile (A)-0.3% Formic acid solution (B), Gradient elution (0~4 min, 5%→40% A; 4~8 min, 40%→65% A; 8~10 min, 65%→85% A; 10~12 min, 85%→5% A; 12~15 min, 5% A); Flow Rate: 1.0 mL/min; Detection wavelength: 254 nm; Column Temperature: 35 °C; Sampling volume: 10 μL.

#### 2.7.2. Preparation of Control

The main internal active ingredients of dried product determined in this experiment were Calycosin-7-glucoside, Calycosin, Ononin, and Formononetin. In the preparation of the mixed control, 3 mg of each component standard was weighed precisely, dissolved and diluted in an appropriate amount of methanol to make a mass concentration of 1 mg/mL of the control. Then diluted step by step to make a stock solution with concentrations of 0.2 mg/mL, 0.1 mg/mL, 0.05 mg/mL, 0.025 mg/mL, 0.0125 mg/mL, 0.00625 mg/mL, respectively. The linearity was examined in terms of peak area (*Y*) against the detection mass concentration (*C*). The regression equation of Calycosin-7-glucoside control was obtained as *Y*_1_ = 46085*C* + 169.41 (R^2^ = 0.9999). The regression equation of Calycosin was obtained as *Y*_2_ = 36016*C* + 203.54 (R^2^ = 0.9999). The regression equation of Ononin was obtained as *Y*_3_ = 32486*C* + 171.57 (R^2^ = 1). The regression equation of Formononetin was obtained as *Y*_4_ = 56153*C* − 60733(R^2^ = 0.9929).

#### 2.7.3. Preparation of Test Samples

Weigh 1.0 g of sample powder in a 50 mL stoppered centrifuge tube, add 25 mL of methanol solution, and then add 25 mL of methanol solution. Ultrasonication in an ultrasonic cleaner (power: 100 W; frequency: 40 kHz; time: 25 min). Centrifugation after sonication (setting parameters: 4000 r/min, 4 °C) for 10 min. The supernatant was filtered through a 0.45 μm filter membrane and then fed into the sample for analysis.

### 2.8. Data Analysis

All tests were repeated three times and the results were expressed as mean values. The obtained data were analyzed by Excel, Origin 18.0 and SPSS 24.0 software.

## 3. Results and Analysis

### 3.1. Analysis of Microwave Vacuum Drying Characteristics of Astragalus

#### 3.1.1. Effect of Drying Temperature on the Microwave Vacuum Drying Characteristics of *Astragalus*

**Figure 5:**
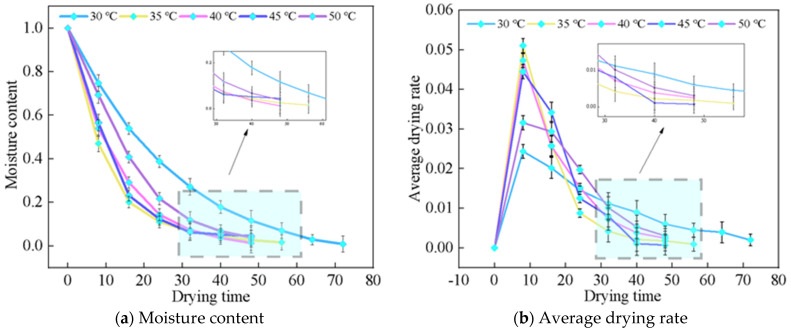
Effects of different temperatures on microwave vacuum drying characteristics of *Astragalus*.

Figure 5 presents the microwave vacuum drying kinetics and rate of material slices at a thickness of 4 mm and vacuum pressure of −0.070 MPa. It can be seen from (a) that the moisture ratio decreases with the increase of drying temperature. Higher temperatures lead to a faster average drying rate and reduced drying time. This pivotal effect asserts a strong positive impact of increased temperature on shortening dehydration time. Observe that (b) found that the curve initially rises before declining. This behavior is due to the internal water of the sample contains more free water in the early stage of drying. The high dielectric constant of free water allows it to absorb microwave energy effectively, resulting in intense molecular friction and collisions that generate additional heat, thereby accelerating water evaporation. As the temperature rises, the vigorous molecular collisions and increased heat absorption further enhance the evaporation process. Consequently, intense molecular collisions and heat absorption occur, leading to increased evaporation. The process then transitions to a deceleration stage (dominated by internal diffusion), as observed. A noteworthy point is that at 50 °C, the drying time is approximately one-third that at 30 °C. Probably with the increase of temperature, the steam inside the *Astragalus* produced a huge driving force, so that water in the form of water molecules moved to the surface and formed a huge pressure difference, prompting the internal water diffusion to the surface, and enhancing mass transfer rates.

Furthermore, the direction of heat transfer within the microwave field aligns with the internal temperature gradient, further increasing the drying rate and reducing overall drying time.

#### 3.1.2. Effect of Slicing Thickness on the Microwave Vacuum Drying Characteristics of *Astragalus*

**Figure 6:**
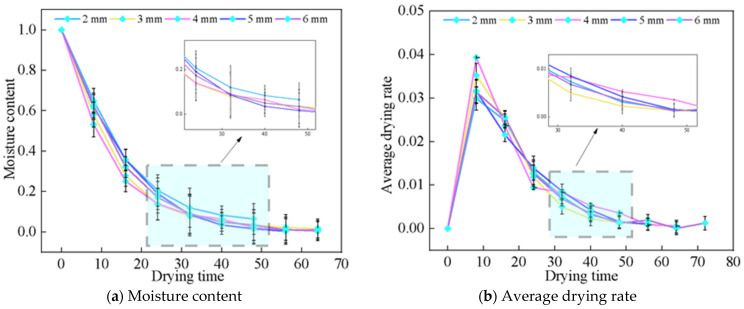
Effect of different slice thickness on microwave vacuum drying characteristics of *Astragalus*.

Visual inspection of Figure 6 clearly illustrates the distinction in moisture ratio and drying rate profiles across varying slice thicknesses. The moisture ratio showed a more consistent behavior, maintaining a steady slope, whereas the drying rate exhibits significant fluctuations, reflecting the intensified water removal dynamics. When the thickness of *Astragalus* slices was 4 mm, the drying rates of the ascending and descending sections responded to more obvious differences. This phenomenon corresponds to the rapid moisture loss, which makes its internal structure change, mirroring Liu’s findings [28]. With the increase of slice thickness, the drying time of the material is positively correlated with the slice thickness of the material. At 6 mm, the longest drying time is observed, demonstrating that thick materials contain more moisture compared to thin materials, rendering each water molecule more resistant to microwave energy absorption and reducing permeability, thus prolonging the drying process. In summary, the drying curve showed that the thinner *Astragalus* tablets evaporated water more rapidly due to their efficient exposure to microwaves. In contrast, thicker slices exhibited a slower drying rate due to less water absorption and heat conduction problems. This highlights significant individual differences in drying time and rate when processing materials. This observation provides important insights and operational recommendations for improving drying processes, such as regulating temperature and humidity, to enhance moisture removal efficiency.

### 3.2. Analysis of Simulation Results

#### 3.2.1. Effect of Microwave on Sample Temperature

**Figure 7:**
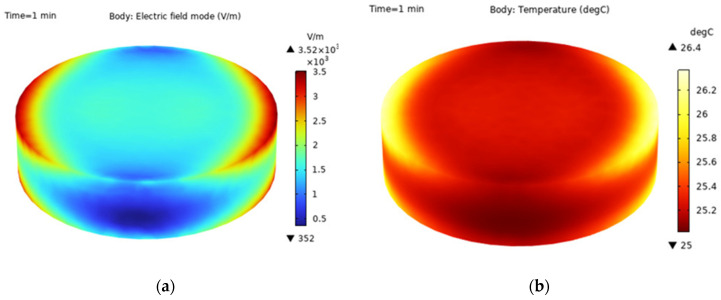
Electric field distribution and temperature distribution at 1 min of drying. (**a**) is the electric field at 1 min of the drying process; (**b**) is the temperature of the drying process for 1 min.

The electric field distribution in the Astragalus sample exhibits considerable variability, as evidenced by a maximum peak of 3520 V/m and a minimum of 352 V/m, highlighting its uneven distribution across various regions. It can be seen that the electric field distribution across various points within the sample is strikingly uneven. Figure 7 illustrates this point: the sample exhibits a peak temperature of 26.4 °C and a minimum of 25 °C. This indicates that the temperature distribution is also notably inconsistent throughout the sample. In more precise terms, regions with higher electric field intensity cause the *Astragalus* sample to heat up faster due to the increased absorption of electromagnetic energy there.

#### 3.2.2. Study on Heat and Mass Transfer Mechanism of *Astragalus* Microwave Drying

**Figure 8:**
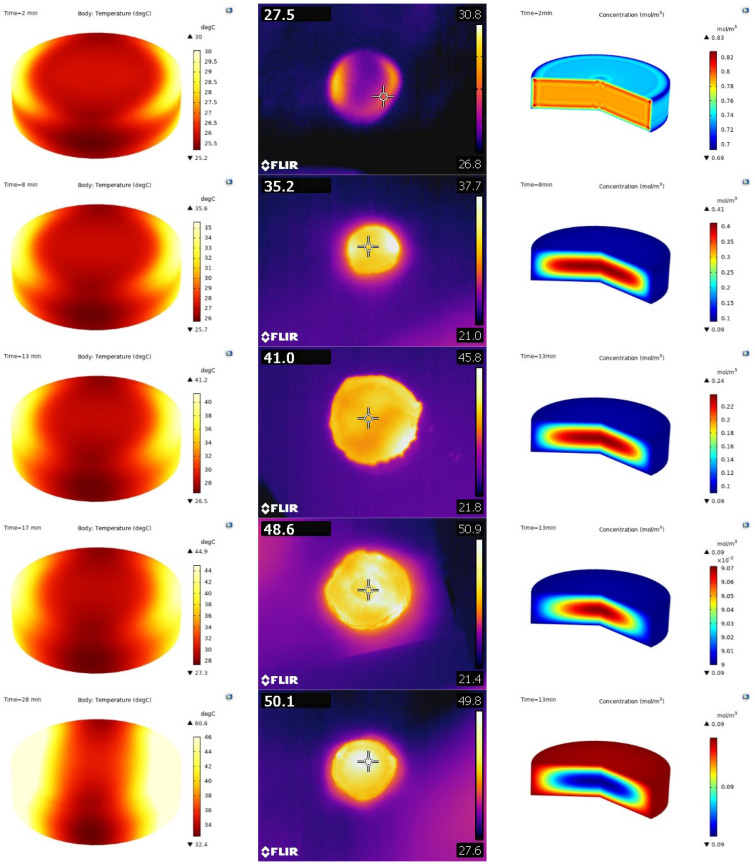
Numerical simulation of microwave drying process of *Astragalus*.

When conducting an analysis of the temperature and humidity changes within *Astragalus* during microwave drying using the COMSOL finite element analysis software, the simulation parameters were set as follows: a temperature of 60 °C, a microwave power of 1500 W, and a microwave frequency of 2.45 GHz. By simulating the intricate drying process (as depicted in Figure 8), it can be seen that the temperature of different regions in the sample is different, which may be due to the uneven distribution of electric field in different regions of the microwave cavity during microwave heating. In areas where the electric field intensity is higher, the temperature also rises accordingly. To verify these numerical predictions, an infrared thermal imager was utilized to measure the temporal temperature profiles of samples, we found that the temperatures measured in the experiment basically match the temperatures predicted by the simulation. Through the simulation of the internal moisture of materials in different time periods, we observed that the internal moisture is higher than the external moisture in the early stage of drying. As the heating process continues, the internal moisture gradually diffuses towards the outer surface of samples. Ultimately, at the end of the drying, the internal moisture concentration had dropped below that of the external, adhering to the fundamental principles of electromagnetic heating, which stresses a balance between heat generation and moisture evaporation.

### 3.3. Analysis of Quality Indicators

#### 3.3.1. Polysaccharide

**Figure 9:**
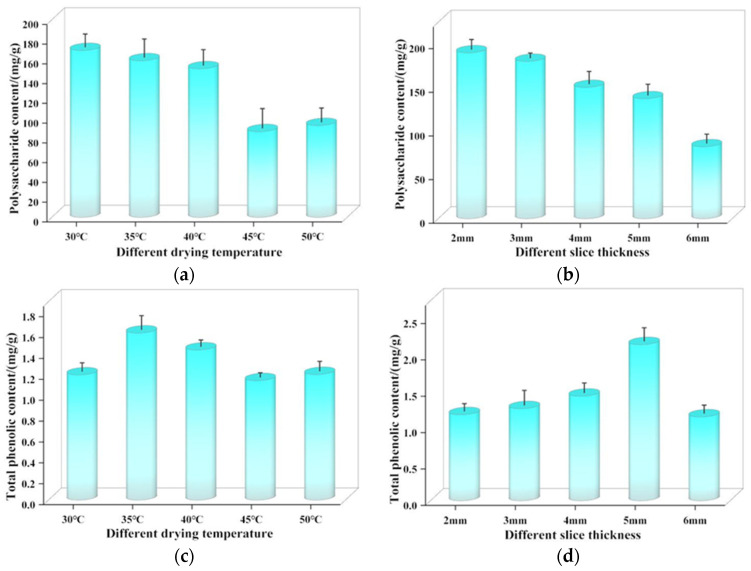

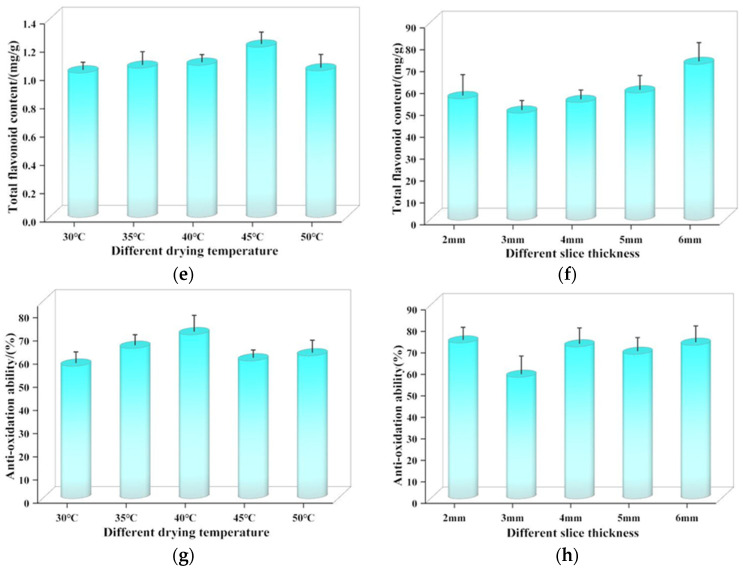
Effects of different drying conditions on polysaccharides (**a**,**b**), total phenols (**c**,**d**), total flavonoids (**e**,**f**) and antioxidant activity (**g**,**h**).

The effects of different drying conditions on the polysaccharide content are shown in Figure 9a,b. It can be clearly observed that the content of polysaccharide decreased first and then increased with the increase of temperature. At 30 °C, the peak amount of polysaccharide is recorded at 168.96 mg/g, while at 45 °C, the minimum value of 86.81 mg/g is observed. This decrease could be attributed to the increase in temperature, which accelerated the rate of the polysaccharide Maillard reaction and severely caramelized it, causing a decline in polysaccharide content. The content decreases with the increase of slice thickness when the temperature is constant. At 2 mm thickness, the highest content, 190.05 mg/g, is observed, while at 6 mm, the figure drops to 82.43 mg/g, a drop of 56.63% from the thicker slices. This may be due to the longer drying duration and higher temperature provided more opportunities for the cleavage of glycosidic bonds in the polysaccharide chain and accelerated its decomposition into oligosaccharides or monosaccharides [29]. To sum up, the modulation of temperature and thickness of *Astragalus* slices significantly impacts its polysaccharide content. At a confluence of 30 °C and a thickness of 2 mm, an optimal balance seems to be achieved, preventing excessive degradation while preserving a reasonable accumulation. As a result, these circumstances hold significant potential for refinement in actual drying operations.

#### 3.3.2. Total Phenols

The effects of different drying conditions on the total phenol content are shown in Figure 9c,d. With the increase of temperature, the total phenolic content showed a trend of increasing and then decreasing. The total phenol content increased from 1.2 mg/g to 1.6 mg/g at 30 °C~35 °C. This increment is attributed to the thermal degradation of macromolecular compounds into smaller, phenolic-rich fragments that release more hydroxyl groups [30]. Conversely, at 35 °C, the total phenol content was at most 1.6 mg/g, which higher by 40.35% relative to 45 °C (1.14 mg/g). This may be due to the fact that phenolics are highly reactive and chemically unstable. At elevated temperatures, the rate of oxidation and thermal degradation accelerates, causing losses phenolic compounds, and the chemical structure of phenols associated with proteins is changed, making the total phenolic compound content lower [31]. When other conditions remain unchanged, changing the slice thickness, total phenol content increased first and then lower trend. The highest total phenol content of 2.15 mg/g is seen at 5 mm, twice that of 6 mm. This may be due to the fact that with a slice thickness of 5 mm, the internal moisture can absorb the maximum amount of microwave energy emitted by the magnetron, and the material does not overheat or absorb insufficient radiant energy [32], allowing a good retention of the total phenolic compound content.

#### 3.3.3. Total Flavonoids

The effects of different drying conditions on the content of total flavonoids are shown in Figure 9e,f. With all parameters held constant, as temperature was elevated, the values rose. As the temperature increases, the contents of total flavonoids were 1.02 mg/g (30 °C), 1.05 mg/g (35 °C), 1.07 mg/g (40 °C), 1.20 mg/g (45 °C), 1.04 mg/g (50 °C), respectively. Initially, the total flavonoid content showed an upward trend, reaching a peak at 45 °C, which may be due to the destruction of flavonoids under high temperature, particularly under shorter drying times, which contributes to the decreased flavonoid content in the dried products. When varying the slice thickness, the 2 mm slice possessed 13.2% higher content compared to 3 mm. This may be because the moisture gradient is consistent with the direction of heat transfer during microwave drying, which reduces the heat transfer resistance. Thinner slices allow for easier microwave penetration, minimizing heat resistance, and reducing drying time, thus helping to maintain a higher flavonoid content. Simultaneously, we observed an upward trend in the total flavonoid content as the thickness of the slices increased. Specifically, when the thickness of the slices varied between 3 mm and 6 mm, the increase in total flavonoid content was particularly noticeable. At a thickness of 6 mm, the total flavonoid content reached its peak at 1.423 mg/g, which is a significant increase of 46.54% compared to the 3 mm thickness. This finding is consistent with research on *Angelica*, which also indicated that increasing slice thickness during the drying process helps to preserve the flavonoid content [33].

#### 3.3.4. Antioxidant Capacity

The effects of different drying conditions on the antioxidant capacity are shown in Figure 9g,h. As temperatures rose, antioxidant capacity showed an upward trend, peaking at 40 °C. This may be at low temperature conditions water content below the safe water content for a long time, leading to a decrease in total phenolic content and weakening the cell’s protective ability against oxidative damage. As the temperature increases, the microwave energy absorbed by the material increases, which intensifies the occurrence of the Maillard reaction during the heating process and generates intermediate products with antioxidant activity [34]. However, when the temperature exceeds 40 °C, the overheating phenomenon of the material leads to the degradation of antioxidant substances, and the antioxidant capacity decreases accordingly. In addition, when temperature varied while keeping other conditions constant, it was found that the antioxidant capacity was similar to the trend of total phenol content. Other studies have also reported a high positive correlation between phenolic compounds and antioxidant activity. Under different slicing thicknesses, the antioxidant capacity does not change much, with values of 72.5%, 56.5%, 70.7%, 67.2%, and 71.4%, indicating that the impact of slicing thickness on antioxidant capacity is relatively small.

#### 3.3.5. Active Ingredients

**Figure 10:**
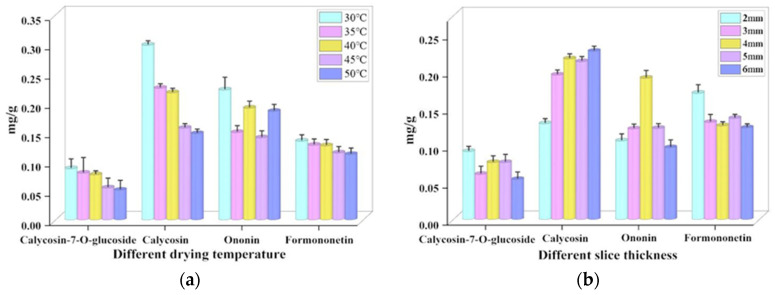
The effects of different drying conditions on the active ingredients. (**a**) represents the effect of different temperatures on the active ingredients; (**b**) represents the effect of different slice thickness on the active ingredients.

The effects of different drying conditions on the active ingredients are shown in Figure 10. A noteworthy observation is that at constant conditions, the highest concentration of all four active ingredients was recorded at 30 °C, with 0.0871 mg/g, 0.2978 mg/g, 0.2214 mg/g and 0.1341 mg/g, respectively. The content of active ingredients showed a decreasing trend with the increase in temperature. Indicating the positive effect of low temperature on the retention of active ingredients in the microwave vacuum drying process. This could be due to Astragalus being under low-pressure conditions where oxygen in the drying medium is scarce, and chemical reactions caused by heat and oxidation are minimal, thus preserving its active ingredients. From (a), it is evident that the effect of temperature on Calycosin content was significant. Its content at temperature 50 °C is 0.15 mg/g, which is half of the maximum content. It may be due to its multiple isomerization reactions under high temperature, making the content decrease. When analyzing the effects of different slice thickness on active components, the retention of Calycosin-7-glucoside and Formononetin decreased, while the content of Calycosin increased, and the content of Ononin increased first and then decreased with the increase of slice thickness. It shows that the thicker the thickness is not conducive to the retention of the two active components of Calycosin-7-glucoside and Formononetin, and has a positive effect on the retention of Calycosin content.

#### 3.3.6. Microstructure

**Figure 11:**
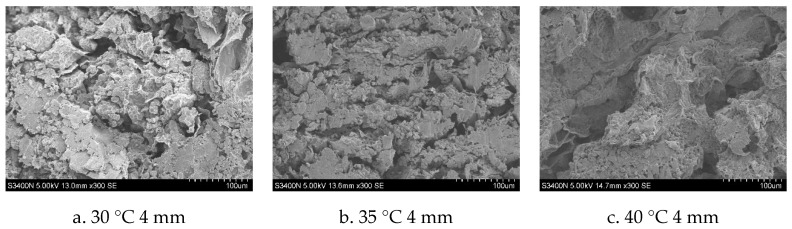

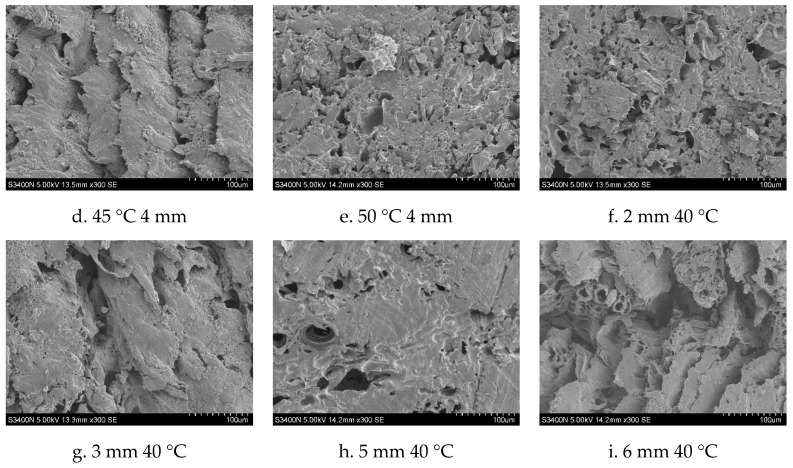
Microstructure of *Astragalus* under different drying conditions.

The surface microstructure of dried productsunder different drying conditions is shown in Figure 11. Its surface is a loose and porous structure. After different drying conditions, the pores and structures of the sample have changed to varying degrees. This phenomenon may be due to the stress caused by the increase of capillary pressure in the material during the drying process [35]. The number of pores on the surface of samples was different at different stages of microwave drying. Higher drying temperatures result in significant cellular contractions, deformations, and fractures within the material, largely due to the rapid loss of moisture and consequent reduction in internal pressure, which leads to surface hardening. This process creates concentrated stress points, causing extensive damage to the tissue structure. When the temperature is 50 °C and the slice thickness is 2 mm, the pore distribution of the surface structure of *Astragalus* is relatively uniform, and there is no obvious crack. This phenomenon can be attributed to the shorter drying time under the aforementioned conditions, as opposed to the less efficient microwave energy absorption observed in other scenarios. When the material absorbs more microwave energy, it will lead to larger pores and more obvious surface structure fragmentation and collapse. These findings align with the research outcomes of Ricardo’s [36]. Under other conditions, microwave vacuum drying typically incurs a prolonged period, leading the material to absorb more microwave energy. This results in a large amount of steam generated during the drying process, increasing the internal pressure gradient, and collapsing the internal structure of the cell [37]. At the same time, as the rapid evaporation of a significant volume of water occurs, the porosity of the material drastically improves, weakening the connections among cells and significantly derailing the microstructure of *Astragalus* [38].

## 4. Conclusions

The effects of microwave vacuum drying on the drying characteristics, quality and microstructure of *Astragalus* were investigated. By analyzing the change pattern of drying characteristics curve of microwave vacuum drying under different slice thickness and temperature conditions, it was discerned that the drying temperature significantly impacts the drying process more than the slice thickness. Among the investigated conditions, slice thickness exhibited a greater overall significance over drying temperature in affecting *Astragalus’s* quality. The mechanism of heat and mass transfer shows that the microwave drying process conforms to the principle of electromagnetic heating. With the increase of temperature, polysaccharide levels initially decreased, followed by increases in total phenolic content, flavonoids, and antioxidant capacity, before eventually declining. With the increase of slice thickness, polysaccharide content decreased, total phenol content increased first and then decreased, total flavonoids and antioxidant capacity decreased first and then increased. Low temperature during microwave vacuum drying had a positive effect on polysaccharide content and the retention of the four active ingredients studied in this experiment. Observing the microstructure of the sample under different drying conditions, it can be seen that the surface micropores are uniform, which is beneficial to the heat transfer of the material. Compared with other microwave drying results of *Astragalus*, this study further revealed the effects of different microwave drying conditions on *Astragalus*. Mainly reflected in the retention of active ingredients. On the whole, in the process of rotary microwave vacuum drying, when the drying temperature is 40 °C and the slice thickness is 4 mm, the physical and chemical quality of dry products is relatively better. It is worth emphasizing that different drying methods were used to study the drying characteristics and quality characteristics of *Astragalus*. However, its thermodynamic characteristics were not considered. On this basis, the water adsorption model of *Astragalus* can be established to predict the change rule of adsorption isotherm under different conditions, and analyze it, so as to further study the thermodynamic characteristics of *Astragalus* in the drying process.

## Data Availability

The original contributions presented in the study are included in the article, further inquiries can be directed to the corresponding author.
